# Progress in Orthopædics

**Published:** 1898-10-29

**Authors:** 


### PROGRESS IN ORTHOPÆDICS.

(*Continued from page* 69.)

**On the Reduction of the Deformity in Pott's Disease.**—Phelps[4] describes this comparatively new method of treatment, and after showing two patients who had been so treated, demonstrated the operation on a third patient. In the first case, with all the force he dared to exert, he could not get the spine perfectly straight. There was no rise of temperature after the operation, the child was up and about in a few days. The application of great force to a tuberculous spine was entirely at variance with the old teaching, which warned us against the danger of using force. This newer, and apparently heroic, method nevertheless resulted in a rapid straightening of the spine, and was attended with little or no reaction. The second patient presented was a boy of five years, who had been first noticed by the mother to have a curvature and a limp about one month before. It was probable, however, that the curvature had existed for at least four months. The limp was due to marked contraction of the leg, yet a careful examination failed to show an abscess burrowing down through the muscle. Partial reduction was obtained under pressure. In commenting upon the operation, Phelps said that no doubt all who witnessed it felt as he had at first, uncomfortable and fearful as to the outcome. He had approached this subject with much hesitation and with many misgivings, and had not entirely gotten over them. The practice, he said, had been in vogue in the time of Hippocrates, and had been revived by Ambrose Pare. Phelps said that Ridlon, who had been the first to do the operation in this country, had operated in seven cases, and had published his results. The operation was certainly not a dangerous one, and paralysis very rarely followed. Further, the operation would certainly relieve paralysis due to bony pressure, and the method seemed to him comparable to that now employed by surgeons for the rapid reduction of the deformity of hip-joint disease. The operation should be avoided in all cases of ankylosed spine with a large curve, also in cases with large abscesses. Good results may be expected in cases of commencing curvature, and in those of recent origin without abscess or in which the abscess had already discharged, and a small curvature remained. Cases of lumbar disease were more favourable than those of dorsal disease, and dorsal more favourable than cervical. He felt sure that the operation would be found a useful one, but its indiscriminate employment by those unable properly to select their cases would probably again relegate it to that oblivion in which it had slumbered for so many years. Dr. Wilson said that he had never been able to convince himself that the breaking down of a curvature, which had been buttressed-up was warranted, but being open to conviction he had attended with the idea of observing the work done in this direction. Whitman pointed out that the point was not whether or not we could straighten the spine in these cases, but whether any permanently good results could be secured. He could not see how there could help being a relapse in cases in which there had been much destruction of bone, but cases of mid-dorsal disease coming under observation in the early stage might, he thought, be greatly benefited in this way. Wyeth thought this operation would be a justifiable experiment in cases of paralysis from pressure due to acute deformity before any changes had occurred as a result of pressure. Such an undertaking seemed to him warranted, though it resulted in injury to the cord. Phelps, in reply, said that he believed, unless the spinous processes were wired, the majority of cases would relapse. If the processes were wired they would rest firmly on the bodies, the two transverse processes and the articular processes; hence, he believed, the wiring would be found necessary to secure permanent results. In the case operated upon that evening he could distinctly feel the deformity give way under his hand.

**Genu Recurvatum.**—Marmaduke Sheild[5] contributes a dissection of one of these interesting cases of congenital genu recurvatum. A child of about eight weeks came under his care. The legs were hyper-extended on the thigh at an obtuse angle, and a transverse furrow extended across the front of the joint as though the popliteal flexors were in front. The feet pointed towards the abdomen. Considerable resistance was offered to any attempt to straighten the limbs, which were otherwise well developed. The hip-joint seemed to be normal, though as the infant had, of course, not walked, it was difficult to estimate whether the acetabulum was well formed. The child was born with a head-presentation. The condition of the limbs was noted at the time. It was a fifth child, and all the others were well formed. The limbs were straightened and flexed under chloroform. About ten days afterwards the child, who looked puny and weak, refused nourishment, and became feverish and died. The cause of death, however, was not clear, and examination of one knee-joint only was made. On dissection the following remarkable conditions were found. The arrangement of the soft parts in the popliteal space was normal, except that the hamstring muscles seemed stretched over the prominent condyles of the femur. The posterior ligament was intact, but somewhat elongated and greatly stretched. The tendon of the quadriceps terminated as usual in the ligamentum patellæ, which had its usual attachment. The position of the bones brought the under surface of the patella to the front of the condyle, above the inter-condyloid notch, and here a very important appearance was noted. The under surface of the patella was not articular, and the back of it was intimately adherent to the front of the femur about the inter-condyloid notch, so that it could only be dissected away with difficulty. There seemed to be no undue contraction of the quadriceps. The crucial ligaments were normal in position, but they resisted flexion, and seemed to be too short. The capsule and lateral ligaments were tight and contracted, and the bones could not be brought into position until these structures were fully divided. The position of the bones was very interesting. The articular surface of the tibia lay on the front of the articular surface of the femur in the position ordinarily occupied by the patella, and the condyles of the femur covered by the stretched posterior ligaments projected remarkably into the popliteal space. The processes of straightening had therefore not really affected the position of the articulations, but the change of position was due to a bending of the tibia at its epiphysial junction. Owing to the position of the patella above the inter-condyloid notch it could not be felt during life, and was taken to be absent.

**Genu Valgum.**—C. A. Morton[6] has taken skiagraphs of six cases of genu valgum, and they all show that there is no rickety curve in the lower end of the femur, and no elongation of the internal condyle, but that the deformity is due to a curvature outward of the tibia, just below the head, associated with a corresponding curve in the shaft of the fibula, which is most marked about the middle of that bone, so that the tibia and fibula at that level are much nearer together than in a normal limb. That this is the essential change in the bones in genu valgum, and not an exceptional one, is shown by the fact that it is present in every one of the six consecutive skiagraphs. In his opinion, the generally received view that the deformity is due to a change in the lower end of the femur is erroneous; at any rate, in regard to the one in young children. He had, therefore, not operated according to MacEwen's method, but had divided the tibia and fibula at the site of the deformity. This paper of Mr. Morton's evoked very considerable correspondence in the medical press, and the matter is still left undecided.

[4] New York Med. Rec., April 23, 1898. [5] Lancet, May 28, 1898. [6] April 16, 1898.

